# ViSwNeXtNet Deep Patch-Wise Ensemble of Vision Transformers and ConvNeXt for Robust Binary Histopathology Classification

**DOI:** 10.3390/diagnostics15121507

**Published:** 2025-06-13

**Authors:** Özgen Arslan Solmaz, Burak Tasci

**Affiliations:** 1 Clinic of Medical Pathology, Elazig Fethi Sekin City Hospital, Elazig 23280, Turkey; 2 Vocational School of Technical Sciences, Firat University, Elazig 23119, Turkey

**Keywords:** intestinal metaplasia, histopathology, transformer networks, ViT, ConvNeXt, Swin transformer, feature selection, iterative NCA, patch-wise analysis, ensemble learning, quadratic SVM, digital pathology, medical image classification

## Abstract

**Background:** Intestinal metaplasia (IM) is a precancerous gastric condition that requires accurate histopathological diagnosis to enable early intervention and cancer prevention. Traditional evaluation of H&E-stained tissue slides can be labor-intensive and prone to interobserver variability. Recent advances in deep learning, particularly transformer-based models, offer promising tools for improving diagnostic accuracy. **Methods:** We propose ViSwNeXtNet, a novel patch-wise ensemble framework that integrates three transformer-based architectures—ConvNeXt-Tiny, Swin-Tiny, and ViT-Base—for deep feature extraction. Features from each model (12,288 per model) were concatenated into a 36,864-dimensional vector and refined using iterative neighborhood component analysis (INCA) to select the most discriminative 565 features. A quadratic SVM classifier was trained using these selected features. The model was evaluated on two datasets: (1) a custom-collected dataset consisting of 516 intestinal metaplasia cases and 521 control cases, and (2) the public GasHisSDB dataset, which includes 20,160 normal and 13,124 abnormal H&E-stained image patches of size 160 × 160 pixels. **Results:** On the collected dataset, the proposed method achieved 94.41% accuracy, 94.63% sensitivity, and 94.40% F1 score. On the GasHisSDB dataset, it reached 99.20% accuracy, 99.39% sensitivity, and 99.16% F1 score, outperforming individual backbone models and demonstrating strong generalizability across datasets. **Conclusions:** ViSwNeXtNet successfully combines local, regional, and global representations of tissue structure through an ensemble of transformer-based models. The addition of INCA-based feature selection significantly enhances classification performance while reducing dimensionality. These findings suggest the method’s potential for integration into clinical pathology workflows. Future work will focus on multiclass classification, multicenter validation, and integration of explainable AI techniques.

## 1. Introduction

Intestinal metaplasia (IM) is a precancerous lesion characterized by the transformation of normal epithelial tissue into intestine-type epithelial cells primarily observed in the gastrointestinal tract, particularly in the stomach and esophagus. This pathological process occurs as a result of chronic mucosal irritation and inflammation and is most commonly associated with conditions such as *Helicobacter pylori* infection, chronic gastritis, autoimmune gastritis, and long-term gastroesophageal reflux [1]. The development of IM involves critical molecular and genetic changes in epithelial cells triggered by chronic inflammation, accumulation of reactive oxygen species, and the release of various cytokines (such as TNF α, IL 1β, IL 8). Key mechanisms in this process include the abnormal expression of transcription factors such as CDX2 and SOX2, as well as the activation of complex molecular pathways like WNT/β catenin, NOTCH, and NF κB signaling [2].

Histopathologically, IM is classified into complete (type I) and incomplete (types II and III) subtypes, with the latter, containing sialomucin, exhibiting the highest malignancy potential. These lesions represent a critical stage in the Correa cascade (normal mucosa → chronic gastritis → atrophic gastritis → IM → dysplasia → adenocarcinoma), a stepwise progression in gastric carcinogenesis. Clinical studies indicate that IM increases the risk of gastric cancer by approximately 6- to 10-fold [3].

Despite available diagnostic methods, some cases of IM may remain undetected due to methodological limitations in biopsy protocols and the sensitivity and specificity of diagnostic tools [4]. Histopathological evaluation can be challenging when goblet cells are scarce, show atypical morphological features, or when IM presents as incomplete or intermediate subtypes, complicating diagnosis with H&E staining [5]. Special histochemical stains (e.g., Alcian blue PAS) are useful for highlighting goblet cells and mucus content, but interobserver variability remains a significant issue [6]. Immunohistochemical techniques, such as markers for intestinal differentiation (CDX2, MUC2), offer additional advantages, but have their own limitations, including heterogeneous expression and reduced diagnostic sensitivity in early-stage lesions [7,8,9]. These molecular and histochemical analyses also impose additional economic costs and require significant laboratory resources. Consequently, even when all available methods are employed, there remains a risk of overlooking IM. This underscores the necessity of a multidisciplinary approach that integrates clinical and endoscopic risk factors with pathological evaluation [10].

Given the diagnostic limitations inherent in conventional histopathological evaluation, the integration of deep learning models has emerged as a transformative approach in medical image analysis. In this study, we propose ViSwNeXtNet, a novel patch-wise ensemble architecture that combines the complementary capabilities of vision transformer (ViT) and ConvNeXt to achieve robust binary classification of histopathology images. By aggregating discriminative features from local image patches, ViSwNeXtNet enhances sensitivity to subtle histological variations that are often difficult to detect through standard methods. This architecture improves classification accuracy while reducing interobserver variability and diagnostic uncertainty. The proposed model demonstrates significant promise as a decision support tool in the pathological diagnosis of IM, with potential implications for early intervention and optimized clinical workflows in gastrointestinal oncology.

### 1.1. Literature Review

In recent years, artificial intelligence (AI) has become an increasingly valuable tool in the field of digital pathology, offering new opportunities to support and enhance diagnostic accuracy. With the growing availability of histopathological image datasets and advancements in computational power, deep learning models, particularly convolutional neural networks (CNNs) and more recently ViTs, have shown considerable promise in detecting and classifying various gastrointestinal lesions. While these approaches have achieved notable success in conditions such as colorectal cancer and gastric adenocarcinoma, the application of AI to the detection of IM remains relatively limited. This is largely due to the subtle, often heterogeneous nature of IM-related tissue changes, which can challenge even experienced pathologists. As a result, researchers have begun to explore ensemble strategies and patch-wise analysis methods that aim to capture fine-grained histological patterns more effectively. In this section, we provide an overview of the current literature on AI-assisted detection of IM, highlighting key methodologies, reported outcomes, and existing gaps that our proposed approach seeks to address.

Gao et al. [11] proposed MSFA Net, a U Net-based model enhanced with multiscale feature fusion and adaptive attention for segmenting precancerous gastric lesions. Using the PLGC hyperspectral image dataset covering IM and GIN stages, the model incorporates CSFF and AWA modules to improve segmentation accuracy. It achieved 92.94% accuracy, 90.90% Dice, 86.46% IoU, and 91.17% precision for IM and 88.12% accuracy, 86.76% Dice, 78.73% IoU, and 85.39% precision for GIN. Cao et al. [12] proposed BE Net, a boundary-aware segmentation network guided by information entropy for segmenting gastric mucosal intestinal metaplasia (IM) and intraepithelial neoplasia (GIN) in microscopic hyperspectral images. Tested on two MHSI datasets containing 412 IM and 282 GIN images, BE Net integrates multiscale spatial and edge features with LoG-based edge extraction and entropy-weighted attention mechanisms. The model achieved 94.14% accuracy, 92.40% Dice, 85.87% IoU, and 93.73% precision for IM and 89.70% accuracy, 89.55% Dice, 81.08% IoU, and 89.43% precision for GIN. Noh et al. [13] conducted a study to identify biomolecular differences between benign (gastritis/intestinal metaplasia) and malignant gastric tissues and to evaluate the diagnostic performance of a Raman spectroscopy-based machine learning approach. A total of 278 Raman spectra were collected in real time during endoscopy from 19 patients in Singapore and analyzed using PCA–LDA with leave-one-out cross-validation. The model achieved 90.5% accuracy, 94.2% sensitivity, 78.7% specificity, and an AUC of 95.7% in distinguishing adenocarcinoma. Fang et al. [14] conducted a study to develop a semisupervised deep learning algorithm for the diagnosis and grading of gastric atrophy and intestinal metaplasia. The study utilized 2725 whole slide images collected from 545 patients suspected of atrophic gastritis across 13 hospitals. A multiscale, transformer-based method called GasMIL was applied, incorporating weakly supervised learning to predict pathology grades without the need for detailed manual annotations. The model achieved strong results on the external test set, with an AUC of 88.4%, sensitivity and specificity of 69%, and a kappa value of 70% for IM and an AUC of 87.7%, sensitivity and specificity of 70%, and a kappa value of 62% for atrophy. One limitation of the study is that severe inflammation and tissue distortion occasionally led to misclassification. Iwaya et al. [15] developed an artificial intelligence system to assess gastric cancer risk by reliably detecting and scoring intestinal metaplasia (IM) using deep learning. The study utilized 5753 whole-slide images from 962 gastric biopsy cases, each containing at least five samples taken in accordance with the Updated Sydney System. A ResNet50-based deep convolutional neural network (DCNN) model was applied for both IM detection and severity scoring. The AI achieved 97.7% sensitivity and 94.6% specificity in distinguishing images with and without IM and 98.5% sensitivity and 94.9% specificity in classifying low-risk (scores 0–1) versus high-risk (scores 2–3) IM. A key limitation is that all data originated from a single institution, limiting the model’s generalizability. Braatz et al. [16] investigated a patch-based sparse whole-slide image classification method for diagnosing gastric intestinal metaplasia (GIM) with a focus on efficiency. They used 318 H&E-stained WSIs (60 GIM positive, 258 negative) from Stanford University and trained EfficientNet B1 models on patch-level annotations. The model achieved an AUC of 98% and average precision of 95%, requiring only 81–164 patches per slide and completing inference in under one minute on a CPU. A key limitation is the use of data from a single institution, which may affect generalizability. Barmpoutis et al. [17] proposed a digital pathology workflow for the segmentation and classification of gastric glands in cases of gastric atrophy and intestinal metaplasia. The study utilized 85 whole-slide images from 20 patients and employed the GAGL VTNet model for gland segmentation and classification. The model achieved an F1 score of 91.4% and a Dice score of 90.8% for gland segmentation, while IM gland classification reached an F1 score of 94%, with 94% sensitivity and 95% specificity. A key limitation is the small sample used in the study. Wang et al. [18] presented a deep learning-based method called W DeepLab to localize and identify intestinal metaplasia in endoscopic images. The model was trained on 200 annotated images from 150 patients and evaluated using semantic segmentation metrics. It achieved 89.5% accuracy and 80.7% mean IoU in detecting lesion areas. Siripoppohn et al. [19] developed a BiSeNet-based deep learning model for real-time detection and segmentation of gastric intestinal metaplasia. Using 802 biopsy-proven images from 136 patients, the model incorporated transfer learning, CLAHE, and data augmentation. It achieved 93% sensitivity, 80% specificity, 86.5% accuracy, and 57% mIoU at an inference speed of 31.5 FPS. A key limitation is the lack of external validation beyond a single-center dataset.

Despite the promising results reported in recent studies, a universally applicable and clinically validated solution for the detection of intestinal metaplasia is yet to be established. The variability in datasets, imaging modalities, and methodological approaches continues to pose significant challenges. These limitations highlight the need for more robust, generalizable, and interpretable models, particularly those leveraging ensemble architectures and patch-wise analysis, to support early and reliable diagnosis. In this context, our proposed ViSwNeXtNet framework aims to contribute to the ongoing effort by offering a novel solution grounded in the strengths of both vision transformers and convolutional architectures.

### 1.2. Novelties and Contributions

This study introduces several key novelties to the field of histopathological image classification for intestinal metaplasia (IM). First, we propose ViSwNeXtNet, a novel patch-wise ensemble framework that combines three complementary transformer-based architectures—ConvNeXt-Tiny, Swin-Tiny, and ViT-Base—to leverage both local and global contextual information from tissue sections. Second, we implement an iterative neighborhood component analysis (INCA) method to select the most discriminative and nonredundant features from a high-dimensional joint feature space (36,864 features reduced to 565), significantly improving computational efficiency and model generalization. Third, our model is evaluated on two datasets: a custom-collected real-world dataset and the publicly available GasHisSDB, demonstrating high accuracy (94.41% and 99.20%, respectively) and robustness across different data sources. Lastly, unlike prior studies that relied solely on end-to-end classification, our modular and interpretable pipeline enables flexible integration into clinical decision-support systems and opens avenues for scalable, explainable AI in pathology. These contributions position ViSwNeXtNet as a strong candidate for real-world application in automated IM detection.

## 2. Materials

**Figure 1:**
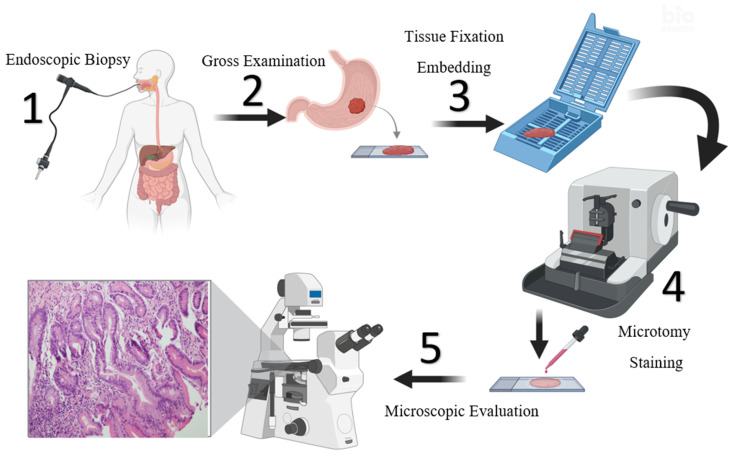
Overview of the histopathological workflow for gastric biopsies. The process includes (1) endoscopic biopsy collection, (2) gross examination, (3) tissue fixation and paraffin embedding, (4) microtomy and H&E staining, and (5) microscopic evaluation for diagnostic confirmation (Microscope’s magnification ×200–×400).

Following approval from the institutional ethics committee, gastric biopsy specimens diagnosed with or without intestinal metaplasia (IM) between January 2023 and April 2025 were retrospectively retrieved from the pathology archives of our hospital. In routine clinical practice, gastric tissue sampling begins with endoscopic biopsy (Figure 1, step 1), followed by gross examination and placement into a cassette for further processing (steps 2 and 3). Tissue fixation and processing were carried out using a short-duration (4 h) automated tissue processor (Leica ASP 300S, Leica, Wetzlar, Germany), after which the samples were embedded in paraffin blocks. Thin sections of 4 μm thickness were cut from these blocks using a microtome (Figure 1, Step 4) and placed on glass slides. Hematoxylin and eosin (H&E) staining was then performed using an automated staining system (Leica Autostainer XL ST5010, Leica, Wetzlar, Germany), with Harris hematoxylin applied for 3 min and 40 s using a regressive staining method. All stained slides were examined under a light microscope (Leica DM 2000, Leica, Wetzlar, Germany) to confirm the presence or absence of IM (Figure 1, Step 5). Following reevaluation, 516 cases with confirmed intestinal metaplasia and 521 control cases without IM were included in the final dataset. Digital images of the H&E-stained slides were captured using an Olympus digital microscope camera for subsequent computational analysis. The histopathological processing steps followed in this study are summarized in Figure 1.

### 2.1. Collected Dataset

This study was conducted using histopathological data collected from patients who applied to Elazığ Fethi Sekin City Hospital between January 2023 and April 2025. A total of 1037 gastric biopsy samples were retrospectively reviewed and included in two categories: 516 cases with confirmed intestinal metaplasia (IM) and 521 control cases without IM. For each patient, a single representative image was selected and used for analysis. For each case, a single representative H&E-stained image was selected under ×200–×400 magnification for analysis.

Among the IM group, 206 were male (mean age: 55.24 ± 14.71) and 310 were female (mean age: 51.22 ± 13.99). In the control group, 209 were male (mean age: 51.85 ± 16.04) and 312 were female (mean age: 49.62 ± 15.38). All biopsy samples were processed using a standardized histological protocol and stained with hematoxylin and eosin (H&E).

**Figure 2:**
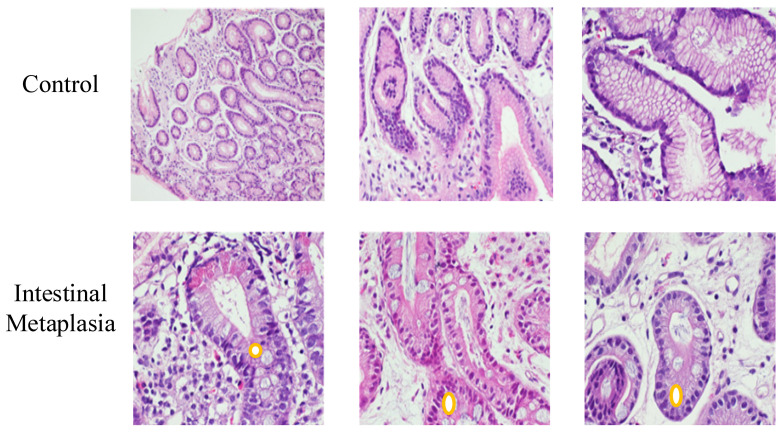
Representative histopathological H&E-stained images of gastric tissue samples. The upper row shows normal gastric mucosa (control), while the lower row displays features characteristic of intestinal metaplasia, including goblet cell (**O**) presence and glandular architectural changes. Control group samples are shown at ×100, ×200, and ×400 magnifications, respectively. Intestinal metaplasia samples are presented at ×200, ×200, and ×400 magnifications, respectively.

The demographic characteristics, including sex and age distribution, were documented in detail to ensure dataset diversity and to evaluate potential diagnostic bias. All stained slides were digitized and used for subsequent AI-based image analysis. Representative examples of normal and intestinal metaplastic gastric tissues are illustrated in Figure 2.

### 2.2. GasHisSDB Dataset

**Figure 3:**
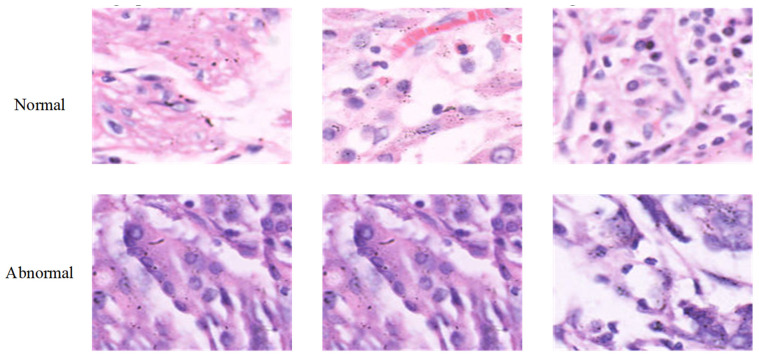
Sample image patches from the GasHisSDB dataset. All samples were visualized at ×400 magnification.

In this study, we employed the open access Gastric Histopathology Subsize Image Database (GasHisSDB), which was developed to facilitate computer-aided diagnosis in gastric pathology [20]. Among its sub-databases, sub-database A, comprising images of 160 × 160 pixels, was used for model development and evaluation. A total of 20,160 images were labeled as normal and 13,124 images abnormal based on histopathological examination of H&E-stained sections. The abnormal class included regions exhibiting morphological features consistent with gastric cancer or its precursors, while the normal class consisted of histologically unremarkable tissue. All images are provided in PNG format and have undergone preprocessing procedures including random rotation and shuffling to minimize redundancy. Samples of normal and abnormal histopathological image patches from the GasHisSDB dataset are shown in Figure 3.

## 3. Our Proposals

In this study, we introduce a novel hybrid deep learning framework named ViSwNeXtNet designed to improve the classification accuracy of intestinal metaplasia (IM) in histopathological images. The proposed method combines patch-wise feature extraction, ensemble deep models, and feature selection to achieve robust binary classification of gastric tissue as either healthy or intestinal metaplasia.

**Figure 4:**
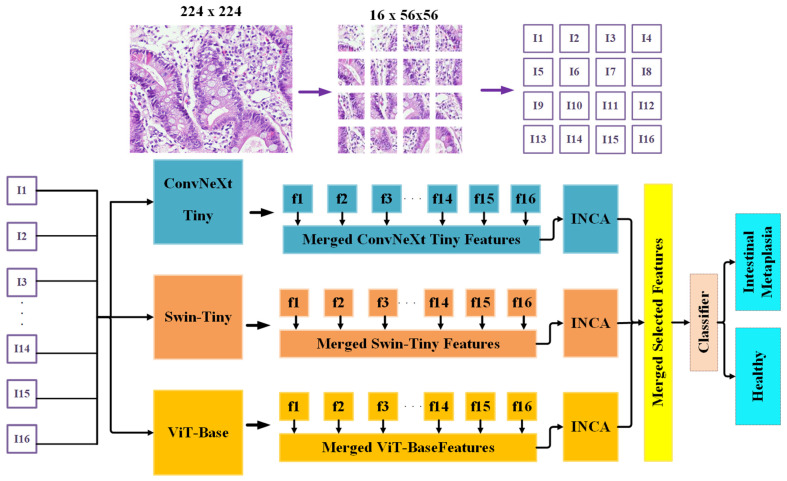
Schematic depiction of the proposed ViSwNeXtNet.

The overall pipeline of ViSwNeXtNet is illustrated in Figure 4. The method consists of the following steps.

**Step 1:** Each histopathological image is resized to 224 × 224 pixels and then partitioned into 16 nonoverlapping patches of size 56 × 56 pixels. This patch-wise strategy ensures that local morphological features are preserved and independently analyzed.

**Step 2:** All image patches are normalized and converted into tensor format suitable for input to deep learning models.

**Step 3:** Each patch is separately passed through three pretrained transformer-based models—ConvNeXt-Tiny, Swin-Tiny, and ViT Base—with their classification heads removed. These models act as fixed feature extractors to obtain deep representations of local tissue structure.

The proposed ViSwNeXtNet framework integrates three distinct transformer-based architectures—ConvNeXt-Tiny, Swin-Tiny, and ViT Base—to extract robust and complementary deep features from histopathological image patches. These models were utilized as fixed feature extractors by removing their classification heads. The architectural details and design principles of each model are described below.

### 3.1. ConvNeXt-Tiny

ConvNeXt-Tiny is a modern convolutional neural network architecture proposed by Liu et al. in 2022 [21] that incorporates several design elements inspired by vision transformers while maintaining a fully convolutional structure. The architecture is composed of a stem layer followed by four sequential stages (ConvNeXtStages), each comprising multiple ConvNeXtBlocks. Each block includes depthwise convolution, LayerNorm, GELU activation, and a two-layer MLP with dropout regularization. In total, ConvNeXt-Tiny contains approximately 50 layers, including convolutional, normalization, MLP, and shortcut layers. The use of LayerNorm instead of BatchNorm, together with the modularity and depth of the architecture, allows it to efficiently capture fine-grained local features. For this study, the classification head was removed and the model was used solely as a feature extractor, enabling effective representation of histological details in tissue images [21].

### 3.2. Swin-Tiny

Swin-Tiny is a hierarchical vision transformer developed by Liu et al. in 2021 [22]. It introduces window-based self-attention mechanisms applied within local nonoverlapping regions, and utilizes shifted windows across layers to capture long-range dependencies. The model architecture consists of four SwinTransformerStages, each containing patch merging layers followed by several SwinTransformerBlocks. Each block combines window-based multihead self-attention, LayerNorm, MLP modules, and residual connections. Swin-Tiny includes approximately 28 layers, accounting for attention heads, feedforward MLPs, and downsampling operations. This hierarchical structure enables multiscale feature representation, making the model particularly suitable for modeling the structural complexity of histopathological tissue architecture [22].

### 3.3. ViT Base

ViT Base, introduced by Dosovitskiy et al. [23], is the first pure transformer-based architecture successfully applied to image classification. The input image is split into fixed-size patches, which are linearly embedded and combined with positional encodings before being passed through a series of transformer encoder blocks. ViT Base contains 12 encoder blocks, each composed of multihead self-attention (MSA), a two-layer MLP, LayerNorm, and residual connections. Including the embedding projection and normalization layers, the model comprises a total of approximately 86 layers. Unlike convolutional models, ViT Base operates globally across the image and excels at learning distributed contextual relationships. In the context of this study, it was utilized to extract global representations from histopathological image patches [23].

**Step 4:** From each model, a 768-dimensional feature vector is extracted per patch, resulting in 16 vectors per image.

**Step 5:** The patch-level features are concatenated for each model, forming a 12,288-dimensional feature vector (16 patches × 768 features) per model per image.

**Step 6:** To reduce redundancy and enhance feature relevance, the high-dimensional feature vectors are independently processed through iterative neighborhood component analysis (INCA), selecting the most informative features from each model.

To address the limitations of traditional neighborhood component analysis (NCA) [24] in selecting redundant features due to its strictly positive weighting scheme, we adopted an iterative NCA (INCA) [25,26] approach to identify an optimal and compact feature subset. Initially, feature vectors were normalized using min–max scaling to ensure compatibility with the distance-based nature of NCA. Feature weights were computed via stochastic gradient descent and ranked in descending order. An iterative loop was then applied, where subsets of features ranging from 100 to 1000 features were incrementally evaluated using a k nearest neighbor (kNN) classifier with Manhattan distance as the loss function. At each iteration, fivefold cross-validation was employed to estimate classification error. The feature subset corresponding to the lowest error was selected as the optimal set. This strategy enables the selection of nonredundant, highly informative features, improving both model efficiency and classification performance. A total of 36,864 features were obtained by concatenating the 12,288-dimensional patch-wise feature vectors extracted from ConvNeXt-Tiny, Swin-Tiny, and ViT Base models. Using the proposed INCA algorithm, this high-dimensional feature set was reduced to 565 optimally selected features, ensuring minimal redundancy and maximal discriminative power. To assess the contribution of these retained features, we performed a permutation-based feature importance analysis on the final 565-dimensional set. This evaluation involved randomly shuffling each feature and observing the resulting accuracy drop in a classifier. The analysis revealed that only a small subset of the retained features had a disproportionately high impact on classification performance, confirming that INCA successfully isolated the most discriminative and nonredundant attributes. This reinforces the effectiveness and explainability of the proposed feature selection approach.

**Step 7:** The selected features from the three models are merged into a single fused feature vector, capturing diverse spatial and contextual information.

**Step 8:** Finally, the fused feature vector is fed into a support vector machine (SVM) [27] classifier, which predicts the image label as either healthy or intestinal metaplasia.

For the classification stage, an SVM with a quadratic kernel function was employed. The kernel scale was set to automatic, allowing the algorithm to determine an appropriate scaling factor based on the data distribution. The box constraint level was fixed at 1, controlling the trade-off between achieving low training error and maintaining a large margin. To handle binary classification, the one vs. one multiclass coding scheme was used, though the task itself remained binary (healthy vs. intestinal metaplasia). Additionally, all input features were standardized prior to training to ensure consistent scaling and improve the stability of the optimization process. This SVM configuration was chosen for its ability to model nonlinear decision boundaries effectively in high-dimensional feature space. All classification results were obtained using 10-fold cross-validation to ensure robustness and generalizability.

The eight steps given above define the suggested ViSwNeXtNet approach.

This multimodel ensemble approach allows the system to leverage both convolutional and transformer-based architectures for comprehensive feature representation. By combining local patch-level information with powerful feature selection and ensemble learning, ViSwNeXtNet addresses key challenges in gastric histopathology, such as subtle morphological variability and class imbalance. Our proposed method demonstrates strong potential as a computer-aided diagnostic tool to assist pathologists in the early and accurate detection of intestinal metaplasia.

## 4. Experimental Results

**Figure 5:**
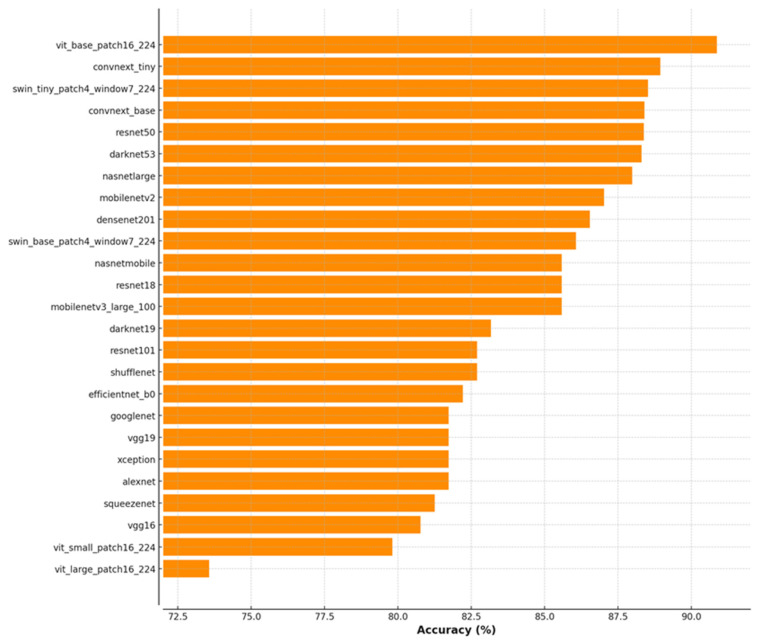
Classification accuracy (%) of 25 pretrained deep learning models using full-sized histopathological images and quadratic SVM. The top-performing models (ViT-Base-Patch16, ConvNeXt-Tiny, Swin-Tiny) informed the architecture of the final ensemble model, ViSwNeXtNet.

Before developing the proposed ViSwNeXtNet architecture, we conducted a comprehensive benchmarking experiment to evaluate the performance of various state-of-the-art deep learning models on our collected histopathological dataset. In this preliminary phase, over 25 pretrained convolutional and transformer-based architectures were used as feature extractors on full-sized (non patched) images, and a quadratic support vector machine (SVM) classifier was applied for final prediction. As illustrated in Figure 5, ViT Base Patch16 achieved the highest classification accuracy (90.87%), followed closely by ConvNeXt-Tiny (88.94%) and Swin-Tiny (88.52%). These findings provided a strong empirical basis for selecting these three models as the backbone of the ViSwNeXtNet ensemble. However, we observed that full-image approaches, though effective, did not fully capture localized morphological variations. Therefore, a patch-wise strategy was adopted in the main ViSwNeXtNet pipeline to enhance sensitivity to fine-grained histological features and improve generalization. The remainder of this section presents the design, implementation, and performance evaluation of the proposed patch-wise ensemble method across two datasets.

**Figure 6:**
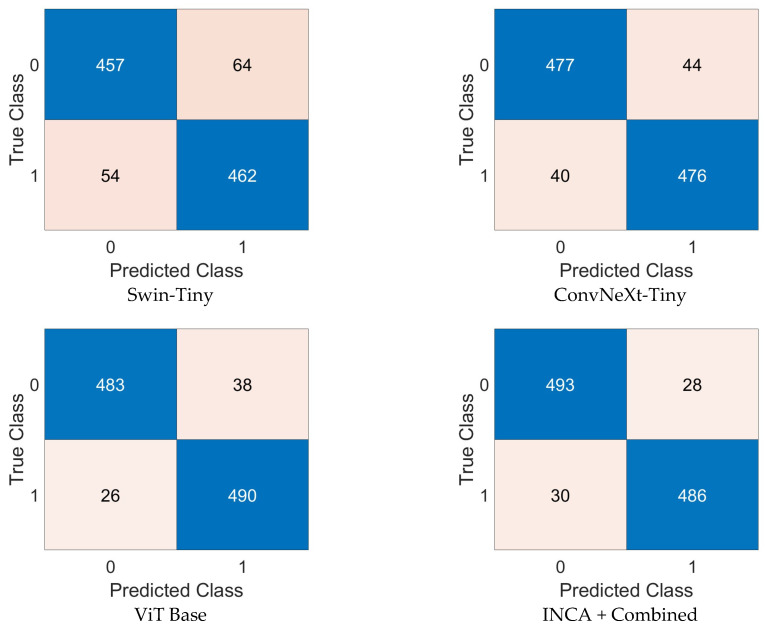
Confusion matrices obtained from the collected dataset using four configurations—Swin-Tiny, ConvNeXt-Tiny, and ViT Base—and the proposed INCA-based combined model (ViSwNeXtNet) (1: intestinal metaplasia; 0: control).

**Figure 7:**
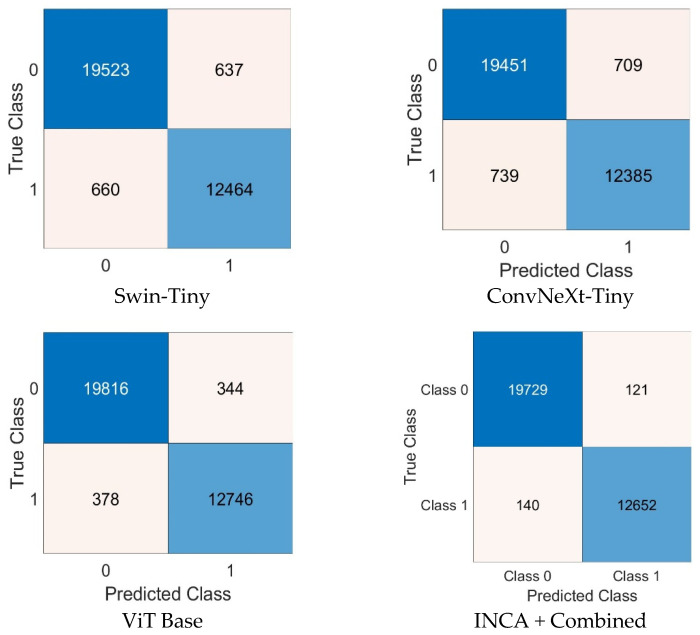
Confusion matrices from the GasHisSDB public dataset showing performance comparisons of Swin-Tiny, ConvNeXt-Tiny, ViT Base, and the INCA + Combined configuration (ViSwNeXtNet) (1: abnormal 0: normal).

The experimental evaluation of the proposed ViSwNeXtNet framework was conducted in two main phases. Steps 1 through 5, which included image preprocessing, patch generation, and deep feature extraction using ConvNeXt-Tiny, Swin-Tiny, and ViT Base models, were implemented in Python 3.11.4 and executed on Google Colab using an Nvidia T4 GPU. Feature vectors of 12,288 dimensions were extracted per model per image, resulting in a combined 36,864-dimensional feature representation for each sample. From step 6 onward, including feature selection and classification, all experiments were performed in MATLAB R2023b. The high-dimensional features were reduced to 565 optimal features using the proposed iterative neighborhood component analysis (INCA). Final classification was carried out using a quadratic SVM classifier, with kernel function set to quadratic, automatic kernel scaling, a box constraint of 1, and standardization enabled. To evaluate the classification performance, we used confusion matrices and standard classification metrics. The confusion matrices generated for the collected H&E dataset are presented in Figure 6, while those for the GasHisSDB public dataset are shown in Figure 7. As can be seen in Figure 6, the individual performances of Swin-Tiny, ConvNeXt-Tiny, and ViT Base yielded moderate results. Specifically, ViT Base achieved the highest accuracy among single models, with minimal false positives and false negatives. The ensemble feature set refined by INCA and classified using quadratic SVM yielded the best performance, with 493/521 correct predictions for the control class and 486/516 correct predictions for the intestinal metaplasia class. In the GasHisSDB dataset (Figure 7), a similar trend was observed. The combined model (INCA + Combined) outperformed individual backbones, achieving 19,729 true negatives, 12,652 true positives, and very few misclassifications. These results demonstrate the advantage of combining multiple feature representations with a robust selection and classification strategy, enabling highly accurate identification of intestinal metaplasia.

Performance metrics computed from the confusion matrices shown in Figure 6 are summarized in Table 1. These include accuracy, sensitivity, specificity, precision, and F1 scores for both the control and intestinal metaplasia (IM) classes. All metrics were calculated based on the standard formulas using true positives, true negatives, false positives, and false negatives derived from the classification outcomes. As shown in Table 1, the performance of the models improves steadily from individual architectures to the combined INCA-based ensemble. The Swin-Tiny model achieved the lowest performance across all metrics, with overall F1 scores of 88.57% for the control class and 88.68% for IM. The ConvNeXt-Tiny model provided a moderate improvement, particularly in precision and specificity. ViT Base outperformed both, demonstrating strong generalization capacity with F1 scores of 93.87% in the IM class and 93.79% in the control class. The highest classification performance was observed with the INCA + Combined configuration (ViSwNeXtNet), which yielded F1 scores above 94% in both classes. Notably, this configuration achieved the most balanced sensitivity and specificity values, indicating minimal class bias and improved robustness. These results validate the effectiveness of combining complementary transformer-based features and refining them through an optimal selection strategy like INCA.

To further evaluate the robustness and generalizability of the proposed ViSwNeXtNet framework, experiments were conducted on the publicly available GasHisSDB dataset. This dataset includes a large number of histopathological images, enabling a more rigorous assessment of model performance in a real-world setting. The confusion matrices resulting from this evaluation are presented in Figure 7, showcasing the classification performance of the individual models Swin-Tiny, ConvNeXt-Tiny, and ViT-Base, as well as the INCA-refined combined model. Corresponding quantitative metrics, including class-wise accuracy, sensitivity, specificity, precision, and F1 score, are provided in Table 2. These results confirm that the proposed feature ensemble and selection approach maintains high accuracy and balanced classification even when tested on an external dataset, thereby validating the robustness of the method.

As illustrated in Table 2, the classification performance of all models improves significantly when applied to the large-scale GasHisSDB dataset, with a clear trend favoring the combined feature strategy. Among the individual models, ViT-Base demonstrates the highest accuracy, reaching 97.83% for the normal class and 97.25% F1 score for the abnormal class, outperforming Swin-Tiny and ConvNeXt-Tiny in nearly all metrics. However, the best overall performance was achieved by the INCA + Combined configuration, with an exceptional 99.2% accuracy, 99.39% sensitivity, and 99.34% F1 score for the normal class, and similarly strong metrics for the abnormal class. These results clearly indicate that the integration of multiple transformer-based feature extractors and the application of INCA for feature selection significantly enhances the classification capability and robustness of the proposed ViSwNeXtNet framework across datasets.

**Figure 8:**
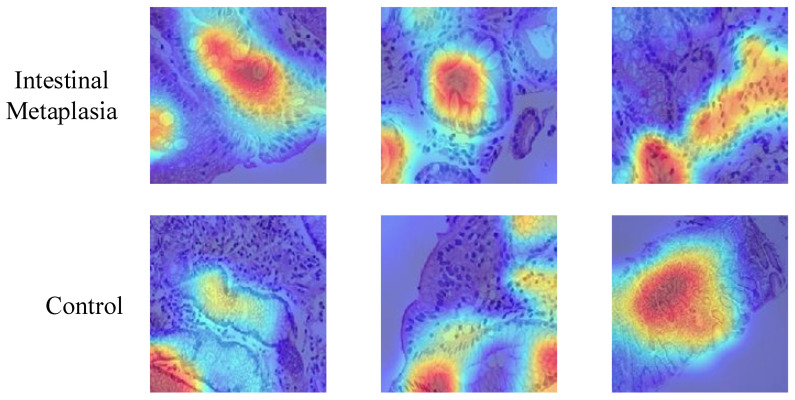
Grad-CAM visualizations of samples. All samples were visualized at ×400 magnification.

To provide insight into the model’s decision-making process, we applied gradient-weighted class activation mapping (Grad-CAM) [28,29,30], a widely used visualization technique that highlights the regions in input images contributing most to the classification outcome. Grad-CAM generates heatmaps by utilizing gradient information flowing into the last convolutional layers, making it useful for interpreting deep learning-based medical image classifiers. As illustrated in Figure 8, the model’s attention in control samples is generally distributed over normal glandular regions, while in intestinal metaplasia cases, it tends to focus on areas with goblet cells and structural alterations. These visualizations support the biological relevance and interpretability of the proposed approach.

## 5. Discussion

Recent advances in artificial intelligence have significantly improved the accuracy of histopathological image classification, particularly in gastrointestinal pathology. Several deep learning and hybrid approaches have been proposed to detect abnormalities such as intestinal metaplasia and early gastric cancer from H&E-stained images. However, many of these models exhibit limitations in generalizability, sensitivity, or computational efficiency. In order to contextualize the performance of the proposed ViSwNeXtNet framework, a comparative analysis of recent state-of-the-art studies is presented in Table 3.

Hu et al. [20] conducted a comprehensive study using the GasHisSDB dataset with both classical machine learning models (SVM, RF) and deep learning architectures such as VGG16, ResNet50, and ViT. While deep models achieved relatively high accuracies (up to 96.47%), their ViT implementation underperformed when training epochs were insufficient, and classical ML models lagged notably behind. Mudavadkar et al. [31] attempted to boost classification performance through ensemble techniques; however, individual models struggled with limited visual extraction, resulting in variable accuracy of 93–98%. Other studies, such as Chen et al. [32] and Song et al. [33], applied transformer-based and CNN-based models to smaller or private datasets. Although high sensitivity was reported, specificity remained moderate in some cases (80.6% in Song et al.), raising concerns about real-world deployment. Zhang et al. [34] explored hyperspectral data, reaching 96.59% accuracy, yet the approach lacks practical scalability in routine pathology labs due to acquisition complexity. In contrast, our proposed ViSwNeXtNet framework, which integrates ConvNeXt-Tiny, Swin-Tiny, and ViT-Base in a patch-wise ensemble structure with INCA-based feature selection, demonstrated strong and balanced performance on both an internally collected dataset and the publicly available GasHisSDB dataset. As summarized in Table 3, ViSwNeXtNet achieved 94.41% accuracy on the collected dataset and an outstanding 99.20% accuracy on GasHisSDB. Furthermore, the method maintained high sensitivity and F1 scores across both datasets, showcasing its ability to generalize across varying sample distributions. Importantly, unlike previous studies that relied solely on end-to-end training or single model outputs, ViSwNeXtNet combines the complementary strengths of multiple transformer architectures and applies rigorous feature refinement. This not only improves robustness but also reduces the feature space from 36,864 to 565 dimensions without compromising classification performance. Therefore, the findings suggest that ViSwNeXtNet represents a promising direction for the development of robust, high-performing computer-aided diagnostic tools in gastric histopathology.

While the proposed model achieved high accuracy on both datasets, some misclassifications were observed. In particular, a subset of intestinal metaplasia cases with weak or atypical goblet cell morphology were incorrectly classified as controls. Similarly, some control cases with mild reactive changes were occasionally misclassified as IM. These failures suggest that the model may struggle with borderline histological presentations and highlight the need for future integration of additional contextual or molecular features to improve robustness.

## 6. Conclusions

In this study, we propose a novel patch-wise ensemble deep learning framework ViSwNeXtNet that combines the strengths of three transformer-based architectures (ConvNeXt-Tiny, Swin-Tiny, and ViT-Base) and integrates iterative neighborhood component analysis (INCA) for optimal feature selection. The method was rigorously evaluated on both a custom-collected histopathological dataset and the publicly available GasHisSDB dataset. Results demonstrated high classification performance in detecting intestinal metaplasia (IM), with the model achieving 94.41% accuracy on the collected dataset and 99.20% accuracy on GasHisSDB, along with high sensitivity and F1 scores in both cases.

The success of ViSwNeXtNet stems from its ability to leverage the local sensitivity of convolutional models, the contextual awareness of window-based transformers, and the global representation capability of vanilla transformers. In addition, INCA significantly reduced the dimensionality of the combined feature set (from 36,864 to 565 features), enhancing computational efficiency without sacrificing accuracy.

For future work, we aim to extend the ViSwNeXtNet framework to support multiclass and multicenter datasets, incorporate explainable AI (XAI) techniques, and evaluate its integration into real-time digital pathology workflows. Additionally, we plan to explore self-supervised or weakly supervised learning strategies to further reduce dependency on large-scale annotated datasets.

## Figures and Tables

**Table 1:** Classification performance metrics of Swin-Tiny, ConvNeXt-Tiny, ViT-Base, and the proposed INCA + Combined (ViSwNeXtNet) model on the collected dataset.

Model	Class	Accuracy (%)	Sensitivity (%)	Specificity (%)	Precision (%)	F1 Score (%)
**Swin-Tiny**	**Control**	88.62	87.72	89.53	89.43	88.57
	**IM**		89.53	87.72	87.83	88.68
**ConvNeXt-Tiny**	**Control**	91.90	91.55	92.25	92.26	91.91
	**IM**		92.25	91.55	91.54	91.89
**ViT Base**	**Control**	93.83	92.71	94.96	94.89	93.79
	**IM**		94.96	92.71	92.80	93.87
**INCA + Combined**	**Control**	94.41	94.63	94.19	94.26	94.44
	**IM**		94.19	94.63	94.55	94.37

**Table 2:** Performance metrics of the Swin-Tiny, ConvNeXt-Tiny, ViT-Base, and INCA + Combined (ViSwNeXtNet) models on the GasHisSDB public dataset.

Model	Class	Accuracy (%)	Sensitivity (%)	Specificity (%)	Precision (%)	F1 Score (%)
**Swin-Tiny**	**Normal**	96.1	96.84	94.97	96.73	96.79
	**Abnormal**		94.97	96.84	95.14	95.05
**ConvNeXt-Tiny**	**Normal**	95.65	96.48	94.37	96.34	96.41
	**Abnormal**		94.37	96.48	94.59	94.48
**ViT Base**	**Normal**	97.83	98.29	97.12	98.13	98.21
	**Abnormal**		97.12	98.29	97.37	97.25
**INCA + Combined**	**Normal**	99.2	99.39	98.91	99.3	99.34
	**Abnormal**		98.91	99.39	99.05	98.98

**Table 3:** State-of-the-art comparison of recent studies on gastric histopathological image classification.

Study	Dataset	Method	Number of Classes	Limitation	Result (%)
**Hu et al. (2022) [20]**	GasHisSDB (245,196 images)	ML (RF, SVM), DL (VGG16, ResNet50, ViT)	2 (normal/ abnormal)	Classical ML models perform poorly; ViT underperforms without enough epochs	ResNet50: 96.09%, VGG16: 96.47%, ViT: up to 94.59%
**Mudavadkar et al. (2024) [31]**	GasHisSDB	Ensemble (ResNet, VGG, EfficientNet, etc.)	2	DL models limited in visual extraction individually	Acc: 93–98%
**Chen et al. (2022) [32]**	Public H&E dataset (280) + 620 images	GasHis Transformer	2	Small dataset; generalizability across stains not fully resolved	Acc: 98.0%, Prec: 98.0%, Recall: 100.0%, F1: 96.0%
**Song et al. (2020) [33]**	2123 WSIs + 3212 real world test slides	ResNet 50, Inception v3, DenseNet	2	Private dataset; specificity moderate (80.6%)	Sens: ~100%, Spec: 80.6%, Acc: 87.3%
**Zhang et al. (2022) [34]**	924 hyperspectral scenes	Symmetrically deep network	2	Traditional pathology tedious; limited early GC detection	Acc: 96.59%
**Our Study**	1037 custom dataset + GasHisSDB (160 × 160)	ViSwNeXtNet (ConvNeXt + Swin + ViT ensemble + INCA)	2 (IM, Normal)		**Collected Dataset** Acc: 94.41% Sens: 94.63% F1 Score: 94.40% **GasHisSDB** Acc: 99.20% Sens: 99.39% F1 Score: 99.16%

Acc: accuracy, Sens: sensitivity (recall), Spec: specificity, Prec: precision.

## Data Availability

The dataset can be downloaded at: https://www.kaggle.com/datasets/buraktaci/histopathology-intestinal-metaplasia (accessed on 2 June 2025).
